# A Non-Cytosolic Protein of *Trypanosoma evansi* Induces CD45-Dependent Lymphocyte Death

**DOI:** 10.1371/journal.pone.0005728

**Published:** 2009-05-29

**Authors:** Nicolas Antoine-Moussiaux, Anne Cornet, François Cornet, Stéphanie Glineur, Martin Dermine, Daniel Desmecht

**Affiliations:** 1 Department of Pathology, Faculty of Veterinary Medicine, University of Liège, Liège, Belgium; 2 Tropical Veterinary Institute, Faculty of Veterinary Medicine, University of Liège, Liège, Belgium; BMSI-A*STAR, Singapore

## Abstract

In a recent study dealing with a mouse model of *Trypanosoma evansi*-associated disease, a remarkable synchrony between the parasitaemia peak and the white-blood-cell count nadir was noticed. The present study was designed to establish whether there is a direct causal link between the parasite load during its exponential phase of growth and the disappearance of peripheral blood leukocytes. *In vitro* experiments performed with trypanosomes and purified peripheral blood mononucleated cells revealed the existence of a lymphotoxin embedded in the *T. evansi* membrane: a protein sensitive to serine proteases, with a molecular mass of less than 30 kDa. Lymphocytes death induced by this protein was found to depend on the intervention of a lymphocytic protein tyrosine phosphatase. When lymphocytes were exposed to increasing quantities of a monoclonal antibody raised against the extracellular portion of CD45, a transmembrane protein tyrosine phosphatase covering over 10% of the lymphocyte surface, *T. evansi* membrane extracts showed a dose-dependent decrease in cytotoxicity. As the regulatory functions of CD45 concern not only the fate of lymphocytes but also the activation threshold of the TCR-dependent signal and the amplitude and nature of cytokinic effects, this demonstration of its involvement in *T. evansi*-dependent lymphotoxicity suggests that *T. evansi* might manipulate, via CD45, the host's cytokinic and adaptive responses.

## Introduction

Trypanosomiasis is a lethal disease affecting both humans and livestock. Human African trypanosomiasis or sleeping sickness is caused by *Trypanosoma brucei rhodesiense* and *T. brucei gambiense*, and animal trypanosomiasis is caused by *T. congolense*, *T. vivax*, *T. brucei brucei*, and *T. brucei evansi*. Lymphopenia is a frequently reported feature of naturally occurring animal trypanosomiases [1], [2], [3], [4], [5]. To our knowledge, and in contrast to mechanisms underlying trypanosomiasis-associated weight loss, anaemia, or mortality, the underlying causes of lymphopenia have not been directly addressed so far. Some available data suggest, however, that lymphopenia can be viewed at least as a marker of trypanotolerance. For example, infection by *T. congolense* of trypanotolerant (West African Dwarf) and trypanosusceptible (Red Sokoto) goat breeds results in lymphopenia only in trypanosusceptible animals [6]. This observation was confirmed later and extended to the trypanotolerant Djallonke sheep breed [7]. Again, upon challenge with *T. congolense*, the CD4+ T-cell and gamma delta T-cell populations decrease in Boran cattle (susceptible) but not in N'Dama cattle (tolerant) [8]. Similarly, among sheep infected by *T. evansi*, the most susceptible animals showed a significant decrease in CD4+ T-cells, whereas self-cured animals did not [9]. Recently, when recording daily changes in the mouse haemogram after infection with *T. evansi*, we observed a remarkable synchrony between the peak of parasitaemia and the nadir of the white blood cell count [10]. The present study was thus designed to establish whether there exists a direct causal link between the parasite load during its exponential phase of growth and the disappearance of peripheral blood leukocytes.

## Materials and Methods

*In vivo* and *in vitro* studies were conducted with leukocytes (peripheral blood mononucleated cells [PBMCs] or splenocytes) purified from blood or spleens from healthy or parasitaemic C57BL/6 mice and phenotyped by flow cytometry. The mice were obtained from Charles River Laboratories. Housing, inoculation, data collection, and euthanasia procedures complied with the NIH guidelines, and the experimental protocol was approved before the study by the Bioethics Committee of the University. The *T. evansi* isolate was obtained from the Institute of Tropical Medicine of Antwerp; it had been isolated in 1997 from a diseased camel by the Agro-Veterinary Institute Hassan II of Rabat (ITMAS #220404A). The parasites were first amplified by a single passage in immune-depressed BALB/c mice (cyclophosphamide, 200 mg/kg body weight). The inoculation procedure consisted of an intraperitoneal (ip) injection of a single 100 µl bolus of a suspension containing 1 to 1.2×10^6^ flagellates per millilitre (as measured with a Neubauer haemocytometre), i.e. ∼10^5^ trypanosomes per mouse. Control mice were mock-inoculated with 100 µl of a suspension containing killed trypanosomes.

### Flow cytometric phenotyping

Leukocytes were labelled with a combination of antimurine CD4-allophycocyanin (rat IgG2a, clone RM4-5, BD Biosciences), CD19-PE-Cyanin7 (rat IgG2a, clone 1D3, BD Biosciences), and CD8-APC-Cyanin7 (rat IgG2a, clone 53-6.7, BD Biosciences), propidium iodide, and annexin V-FITC (Apoptest-FITC™, DakoCytomation). Three microlitres of each antibody were distributed into Eppendorf tubes. Then, 500 µl of fresh leukocytes (total leukocytes, PBMCs, or splenocytes, within 1 h of withdrawal [see below]) suspended in PBS were added, gently mixed, and incubated for 20 min on ice in the dark. Afterwards, the cells were washed with ice-cold PBS and the Apoptest-FITC™ kit was used according to the manufacturer's instructions (15 min). Samples were analysed within 10 min in a FacsCanto (BD Biosciences) flow cytometre. Instrument settings were adjusted using fresh PBMC samples in eight tubes (an unlabelled sample, five single-labelled samples, one isotype-matched nonpertinent control, and one sample labelled with the mixed antibodies) and FACS Diva™ calculation software (BD Biosciences). Detection and compensation stability were tested daily with SetUp™ beads and FACSCanto™ software (BD Biosciences). Dot plots were analyzed with FACS Diva™ (v.5.0.3., BD Biosciences) software. Absolute counts were obtained by multiplying the number of events detected in each subpopulation of events by the volume of the suspension analysed by the cytometer and by normalizing the result obtained to 1 ml mouse blood (taking the dilution factor of the blood into account). The volume of the cell suspension analysed is the product of the duration of the analysis (s) by the sample flow rate (1 µl/s).

### *In vivo* studies

Two successive *in vivo* studies were conducted first. The first aimed at counting and phenotyping circulating leukocytes during the first parasitaemic wave (n = 24 mice, of which 12 controls were mock-inoculated). The second was designed to establish whether observed changes would be altered by neutralization of tumour necrosis factor-α (TNFα) or nitric oxide (NO) (n = 18, of which 6 controls were mock-inoculated). TNFα neutralization was achieved by daily ip injection of 10 µg anti-mouse TNFα mAb (rat IgG1, clone MP6-XT22, Biosource) into each mouse (n = 6) [11]. Endogenous NO synthesis was inhibited in a second batch of 6 mice by supplementing their drinking water with 1 mg/ml N-nitro-L-arginine methyl-ester (L-NAME) [12]. The L-NAME-treated and untreated control groups were mock-injected ip daily with 50 µl PBS. Seven successive blood samples were drawn from each mouse enrolled in each study, three before inoculation (bi) to reveal the normal leukogram (72, 48, and 24 hours bi) and four post-inoculation (pi) during the ascending phase of the first parasitaemic wave, as determined from preliminary studies (52, 64, 82, and 92 hours pi). The blood sampling procedure consisted in collecting a volume of 10 to 15 µl tail blood by capillarity with a heparinized 75-µl micro-haematocrit tube (Hirschmann Labogeräte). Parasitaemia and haematological changes were evaluated by flow cytometry as previously described [10]. For monitoring apoptosis/necrosis in leukocytes, 10 µl heparinized blood was gently mixed with 500 µl red blood cell lysis solution (0.15 M NH_4_Cl, 10 mM KHCO_3_, 0.1 mM Na_2_-EDTA, 1 mM NaN_3_) and leukocytes were harvested by centrifugation (8 min at 300 g and 4°C), washed once in ice-cold PBS with 5% BSA, and suspended in 500 µl PBS for labelling.

After the *in vitro* studies were conducted (see below), a third *in vivo* study was designed to establish whether the <30 kDa filtrate of the non-cytosolic fraction of the parasites would alter leukocyte counts and regressive changes when injected intravenously. Mice were treated iv with 100 µl of either PBS (n = 6) or the semi-purified non-cytosolic fraction (n = 6), the latter corresponding to 4–5×10^9^ trypanosomes per ml of blood. Two successive blood samples were drawn from each mice, the first 30 min before and the second 120 min after the injection and haemogram and leukocyte apoptosis/necrosis changes were monitored as aforedescribed.

### *In vitro* studies

#### Cells

Peripheral blood lymphocytes and other mononucleated cells (PBMCs) were isolated from the pooled blood of a set of healthy donor mice by polysucrose density gradient centrifugation at room temperature (Accuspin™ System Histopaque®-1077, Sigma). After centrifugation the supernatant was discarded and the cell-containing opaque band at the interface between plasma and Histopaque®-1077 was collected carefully. The PBMCs were then washed once with Hank's balanced salt solution (HBSS), centrifuged (300 g, 10 min at room temperature), washed another three times in HBSS, and resuspended in RPMI 1640 medium supplemented with heat-inactivated foetal bovine serum (10%), MEM non-essential amino acids (1%), penicillin (1%), streptomycin (1%), and 2-mercaptoethanol (0.002%) for cell count determination (Neubauer chamber). Then the cell suspensions were adjusted to 10^6^ cells/ml, distributed into Cellstar® 24-well plates (100 µl/well, i.e. 10^5^ cells/well, Greiner Bio-One), and incubated at 37°C under 95% humidity and 5% carbon dioxide. After 30 or 60 min (see below), 50 µl of each well suspension was transferred to BD FACS tubes, diluted 10 times with PBS, and phenotyped with the Apoptest-FITC™ kit prior to flow cytometric detection of necrotic and apoptotic cells. Splenocytes were isolated by passaging minced spleen fragments through a nylon mesh and mixing with red blood cell lysis solution. Afterwards, they were conditioned and analysed like PBMCs.

#### Parasites and parasite fractions

Trypanosomes were isolated by DEAE-cellulose chromatography [13] from the pooled blood of a set of donor mice that had been inoculated 96 hours before exsanguination, i.e. during the exponential phase of parasite growth. Purified parasites were collected in phosphate saline glucose (pH 8), washed, and resuspended in the supplemented RPMI 1640 medium (live parasites) or PBS (killed parasites) for counting (Neubauer chamber). Trypanosome-free plasma from the same donor mice was obtained by centrifuging and filtering (0.2 µm pores) heparinized whole blood. Serial dilutions were made by adding plasma drawn from healthy mice. To generate trypanosome fractions, a suspension titrating 4×10^8^ parasites per ml, obtained by DEAE-cellulose chromatographic isolation, was repeatedly freeze-thawed, thoroughly vortexed until no intact trypanosome could be detected under the microscope, and centrifuged for 30 min at 14 000 g and 4°C to separate the cytosolic and non-cytosolic fractions.

#### Experimental design

Firstly, PBMCs were exposed to fresh trypanosomes and to trypanosome-free plasma purified from the pooled blood of a set of donor mice having been inoculated 96 hours before exsanguination, i.e. during the exponential phase of parasite growth. The stock suspension was adjusted to 10^9^ parasites/ml, and a series of dilutions was made before 150 µl of each was distributed into the above-mentioned PBMC/splenocyte-containing 24-well plates. The dilutions were calculated so as to expose the PBMCs/splenocytes to parasite concentrations approximating those observed during the exponential phase of parasite growth *in vivo*. The PBMCs/splenocytes were thus exposed to the following parasite concentrations (each in triplicate): 0, 4×10^6^, 4×10^7^, 1×10^8^, 2×10^8^, 3×10^8^, 4×10^8^, and 6×10^8^ trypanosomes/ml, corresponding to parasite-to-cell ratios of 0, 10, 100, 250, 500, 750, 1000 and 1500 respectively. In the second study, PBMCs were exposed to live or dead trypanosomes (4×10^8^/ml) and to corresponding cytosolic and non-cytosolic fractions. In the third study, splenocytes were exposed to serial dilutions of the non-cytosolic fraction for 15 to 120 min. In the fourth study, the non-cytosolic fraction was first stored for 24 hours before being added to the splenocyte suspension. Different conditions were imposed during storage: (i) 4 or 37°C, (ii) 4 or 37°C with/without inhibitors of serine proteases (Pefabloc® SC, 1 mM, Fluka), (iii) 37°C with/without inhibition of cysteine proteases (E64, 10 µM, Sigma-Aldrich), (iv) 4 or 37°C with/without proteinase K (5 U/ml, Sigma-Aldrich), and (v) 37°C with/without DNAse I (2000 U/ml, Invitrogen). In the fifth study, the non-cytosolic fraction was sub-fractionated by means of Microcon centrifugal filter devices with cut-offs at 50 and 30 kDa (Millipore). Splenocytes were then exposed for 75 min to the recovered filtrates and retained material. In the sixth study, splenocytes were exposed to the non-cytosolic fractions (complete and both filtrates) for 75 min after addition of phosphatase inhibitor 1 or 2 (Sigma-Aldrich), each at 1∶100 (v/v) dilution, to the incubation mixture. At neutral pH, these inhibitors specifically inhibit serine/threonine or protein tyrosine phosphatases (PTPases), respectively. Next, the phosphatase activity of each fraction was measured with a colorimetric *p*NPP Protein Phosphatase Assay Kit (AnaSpec SensoLyte™). Finally, a CD45 PTPase inhibition assay was carried out, in which splenocytes were exposed to increasing concentrations (1, 1.5, and 2 µg/5×10^5^ cells) of anti-CD45.2 mAb (mouse IgG2a, clone 104-2, Abcam) for 10 min prior to addition of the non-cytosolic fraction. Anti-CD45.1 mAb (mouse IgG2a, clone A20, Abcam), targeting an epitope that is absent in C57BL/6 mice, was used as a control.

### Statistical analyses

A mixed-model analysis was applied to the *in vivo* data in order to test the effect of infection on each dependent value using a combination of autoregressive structures within animals (time points) and a random effect between animals (SAS® software v. 8.2, *Mixed procedure*, SAS Institute Inc. [14]). For *in vitro* studies, the GraphPad Prism software was used for statistical analysis by Student's *t* test. Values of *p*≤0.05 are considered statistically significant.

## Results

### *In vivo* studies

**Figure 1:**
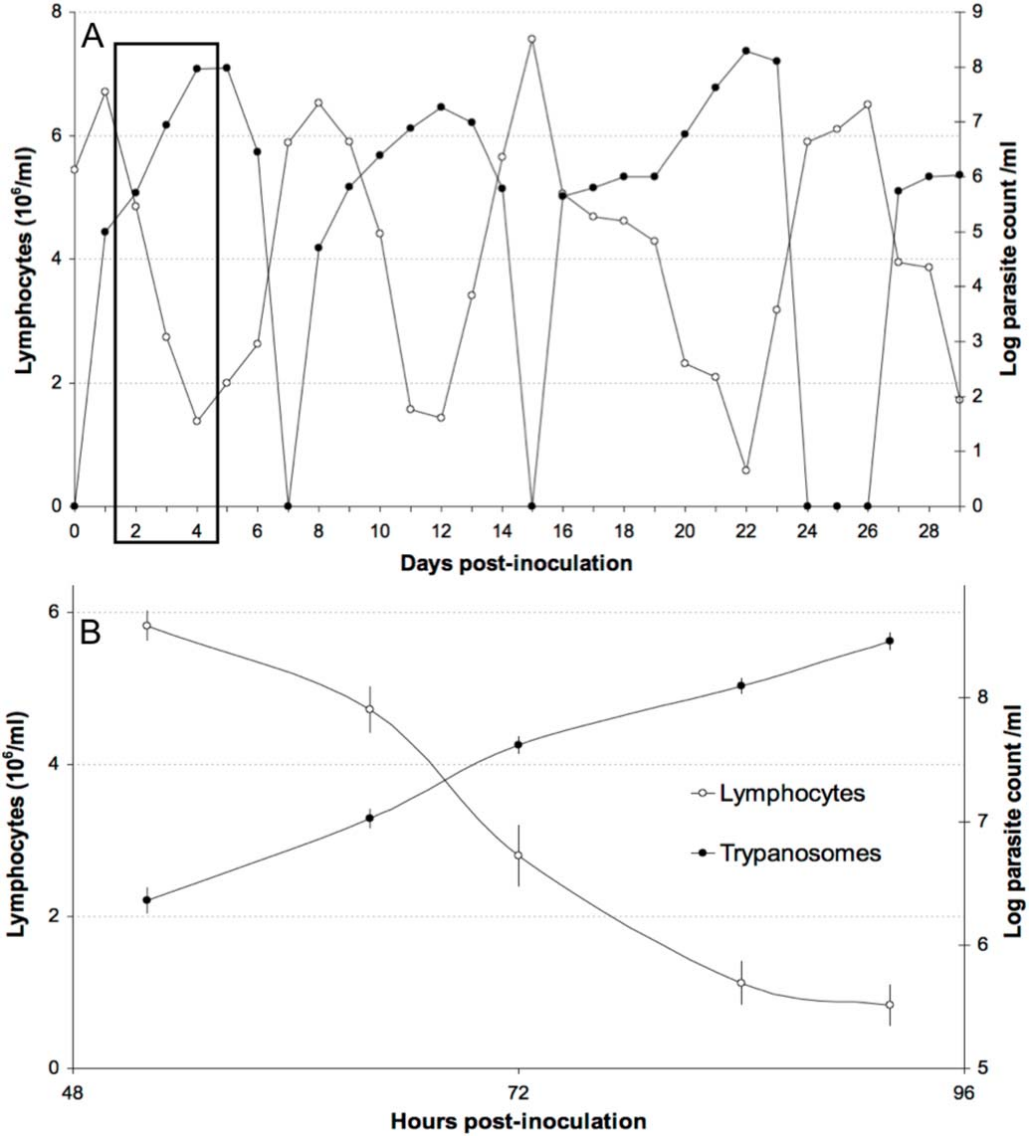
Evolution of peripheral blood parasite and lymphocyte counts in C57BL/6 mice inoculated with *T. evansi*. A: parasite and lymphocyte counts in a representative C57Bl/6 mouse over a 30-day period after *T. evansi* inoculation. Synchrony between successive parasite peaks and lymphocyte troughs is clear. B: enlarged view of the time window during which lymphocyte death was sought *in vivo*.

**Figure 2:**
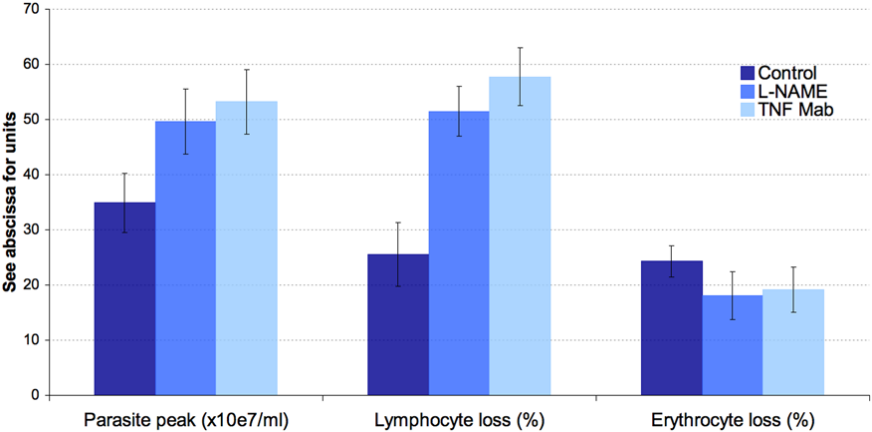
Effect of *in vivo* neutralization of TNFα and NO on parasite, erythrocyte, and lymphocyte counts. Lymphocyte loss was enhanced by neutralization of either TNFα or NO, whereas erythrocyte loss appeared to be slightly reduced by both treatments. The more severe lymphopenia observed in treated animals might be linked to the higher parasite peak resulting from neutralization of either TNFα or NO.

Daily monitoring of parasitaemia and of the circulating lymphocyte count after inoculation of C57BL/6 mice with *T. evansi* revealed successive, regular, antisynchronous waves of parasitaemia and leukopenia (Fig. 1). The first was detected 4–6 days pi. The quantitative values of the parasitaemia peak and leukocyte-count nadir and of the rising and descending slopes were quite reproducible from wave to wave and from mouse to mouse. In a second experiment, total lymphocytes and healthy (annexin V- and PI-negative), apoptotic (annexin V-positive and PI-negative), and necrotic (annexin V- and PI-positive) lymphocytes were counted during the exponential growth phase of the first parasitaemia wave by increasing the number of blood samples taken during the appropriate time window identified in the first experiment (Table 1). Overall, the counts of circulating parasites and lymphocytes were again found to evolve antisynchronously (Fig. 1). Parasitaemia peaked at 2.9×10^8^±2.0×10^7^ trypanosomes per ml blood and the leukocyte nadir was at 13.5±4.2% of the initial count. The LB∶LT ratio was found to increase significantly from the 52nd to the 84th hour pi (p<0.05), i.e. between the beginning and the end of the drop in circulating lymphocytes. The CD4∶CD8 ratio also increased significantly (p<0.05). Furthermore, when the inoculated mice had received an injection of anti-TNFα mAb or when L-NAME had been added to their drinking water, the observed changes were exacerbated (Fig. 2), with a higher parasitaemia peak (p<0.05) and a greater loss of circulating lymphocytes (p<0.0001). The anaemia, on the other hand, was less pronounced. Lastly, healthy and inoculated mice showed no significant difference in their counts of circulating apoptotic and necrotic lymphocytes (p>0.05).

**Table 1:** Parasite load, lymphogram, and lymphocyte death during the first parasitaemia wave.

Time	1 h bi		52 h pi		64 h pi		72 h pi		84 h pi		92 h pi	
	CTL	PRINC.	CTL	PRINC.	CTL	PRINC.	CTL	PRINC.	CTL	PRINC.	CTL	PRINC.
Parasite load (×10^7^/ml)	0	0	0	0.23±0.57	0	1.07±0.20	0	4.17±0.65	0	12.6±1.78	0	28.9±1.96
Lymphocytes (×10^6^/ml)	5.16±0.24	5.01±0.15	5.02±0.21	5.83±0.21*	5.23±0.17	4.72±0.30*	4.99±0.24	2.80±0.40*	5.41±0.26	1.13±0.29*	4.88±0.24	0.83±0.26*
CD4+ (×10^6^/ml)	1.25±0.18	1.24±0.14	1.12±0.12	1.00±0.15	1.11±0.19	0.83±0.14*	1.17±0.13	0.63±0.10*	1.16±0.19	0.24±0.04*	1.10±0.14	0.19±0.05*
CD8+ (×10^6^/ml)	0.69±0.08	0.67±0.08	0.67±0.06	0.62±0.06	0.67±0.08	0.45±0.06*	0.63±0.08	0.25±0.02*	0.58±0.08	0.11±0.02*	0.58±0.06	0.08±0.01*
CD19+ (×10^6^/ml)	3.22±0.26	3.10±0.21	3.23±0.20	4.20±0.20*	3.45±0.25	3.45±0.19	3.19±0.16	1.92±0.10*	3.67±0.25	0.78±0.06*	3.20±0.18	0.56±0.07*
CD4+ (%)	24.3±3.5	24.7±2.9	22.2±2.4	17.2±2.6*	21.2±3.6	17.5±2.9*	23.4±2.6	22.6±3.5	21.4±3.4	21.2±3.5	22.5±2.8	22.5±6.1
CD8+ (%)	13.4±1.6	13.3±1.5	13.4±1.2	10.7±1.0*	12.8±1.6	9.5±1.3*	12.7±1.7	8.8±0.9*	10.7±1.5	9.4±2.1	12.0±1.3	10.1±1.8*
CD19+ (%)	62.3±5.0	61.9±4.1	64.4±3.3	72.1±3.5*	66.0±4.8	73.0±4.1*	64.0±3.1	68.5±3.7*	67.9±4.7	69.5±5.4	65.5±3.6	67.5±8.0
Apoptotic CD4 (%)	5.41±1.35	5.12±1.6	5.64±1.33	6.12±1.44	4.56±1.04	6.74±1.51	4.13±0.82	5.19±1.23	3.25±0.86	5.07±1.34	5.74±0.86	5.28±1.34
Apoptotic CD8 (%)	1.40±0.47	1.70±0.87	1.54±0.58	2.40±1.69	1.56±0.43	1.73±1.23	1.10±0.28	1.15±0.44	1.05±0.51	1.11±0.59	1.32±0.51	1.45±0.59
Apoptotic CD19 (%)	0.39±0.23	0.67±0.37	0.76±0.36	0.69±0.39	0.53±0.34	0.62±0.29	0.41±0.14	0.56±0.27	0.48±0.18	0.45±0.30	0.54±0.18	0.48±0.30
Necrotic CD4 (%)	0.5±0.29	0.6±0.22	0.4±0.09	0.5±0.09	0.4±0.11	0.4±0.15	0.3±0.09	0.4±0.08	0.35±0.19	0.4±0.18	0.4±0.18	0.5±0.11
Necrotic CD8 (%)	0.5±0.44	0.5±0.17	0.3±0.17	0.2±0.07	0.1±0.03	0.2±0.11	0.3±0.13	0.5±0.30	0.2±0.10	0.3±0.10	0.2±0.12	0.5±0.28
Necrotic CD19 (%)	2.0±2.05	1.8±1.02	2.2±0.83	2.0±0.81	3.9±2.61	1.5±0.75	2.0±0.69	1.5±0.82	2.5±0.94	1.9±0.64	2.6±1.08	1.69±0.81

Results are means±SD. CTL, control mice; PRINC., principal mice inoculated with *T. evansi*; bi, before inoculation; pi, post-inoculation. Asterisks show values significantly different from corresponding isotime values among control mice (p<0.05).

### *In vitro* studies

Seven successive *in vitro* studies were conducted, with either PBMCs or splenocytes.

**Figure 3:**
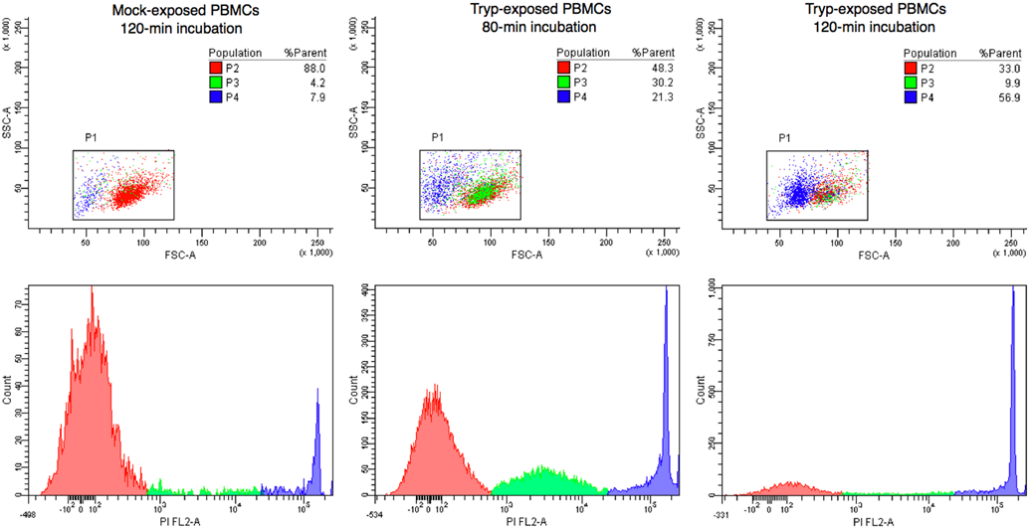
Exposure of mouse PBMCs to trypanosomes results in cell death. Forward (FSC) and side (SSC) scatter dot plots (upper panels) and corresponding frequency distribution of PI incorporation among mock- and killed *T. evansi*-exposed PBMCs. P1 (red): annexinV/PI double-negative PBMCs; P2 (green): annexinV/PI double-positive cells with ∼intact FSC/SSC; and P3 (blue) annexinV/PI double-positive PBMCs with a skewed FSC profile suggesting size reduction. A stepwise process is obvious, with size preservation and moderate PI incorporation first, followed by marked PI incorporation and a drastic size reduction at a later stage. Relative population sizes are shown in the three top panels.

A general observation from flow cytometric analyses is that the FL2/SSC dot plots obtained for mock-exposed PBMCs (Fig. 3) revealed a majority sub-population of PI-negative cells (P1) and two minority sub-populations of PI-positive cells (P2 and P3). Populations P1 and P2 showed identical morphological profiles (FSC/SSC), whereas population P3 displayed a diminished FSC, suggesting a smaller size. As necrosis is a stepwise process in which cells become progressively smaller and more permeable to PI, the three populations most likely correspond to intact cells (P1) and to cells undergoing early (P2) and late (P3) steps of the necrosis process. The fact that a small population of cells undergoing necrosis (2.8±0.3% [P2] and 11.7±0.3% [P3]) was consistently found among mock-exposed control PBMCs probably reflects a deleterious effect of the purification process.

**Figure 4:**
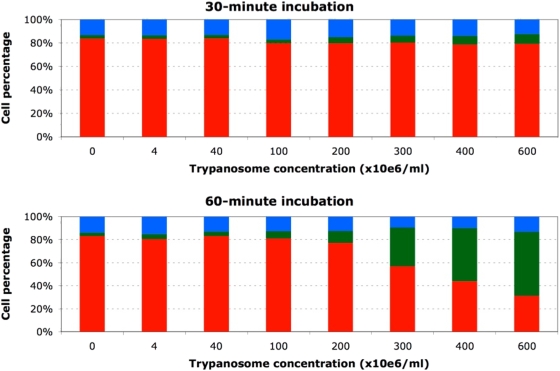
Exposure of mouse PBMCs to live trypanosomes results in time- and dose-dependent cell death. The three cell subsets represented are normocytic and annexinV/PI double negative (red, healthy), normocytic and annexinV/PI double positive (green, early necrosis) and microcytic and annexinV/PI double positive (blue, late necrosis). Values presented are means of three independent experiments.

**Figure 5:**
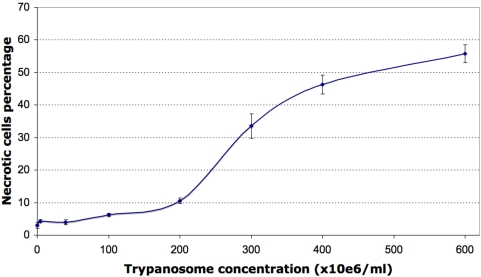
Effect of trypanosome load on PBMCs. The cells show changes typical of early necrosis after a 60-min exposure. Values are means±SD.

In the first study, PBMCs exposed to trypanosome-free plasma from parasitaemic mice yielded similar FL2/SSC and FSC/SSC dot plots (3.1±0.6% [P2] and 9.5±1.1% [P3]), suggesting that the blood of parasitaemic mice contains no soluble factor capable of causing leukocyte necrosis. In contrast, PBMC exposure to a suspension in RPMI of live trypanosomes purified from a pool of blood taken from parasitaemic mice considerably modified the relative sizes of the three populations, increasing significantly the size of populations P2 and P3 and thus suggesting trypanosome-triggered induction of necrosis (Fig. 3). This pronecrotic activity was found to increase with the duration of PBMC exposure to the trypanosomes (Fig. 4). A sigmoid profile was obtained when the relative size of population P2 was plotted versus the quantity of trypanosomes added (Fig. 5), the increase being quasi-linear at parasite-to-PBMC ratios between 500 and 1000. The FL1/SSC dot plots of PBMCs exposed to suspensions of live trypanosomes consistently revealed the existence of a very marginal apoptotic sub-population (PI-negative and annexin-V-positive) not significantly different in size from that found among mock-exposed control PBMCs (5.3±0.8%, p>0.10).

**Figure 6:**
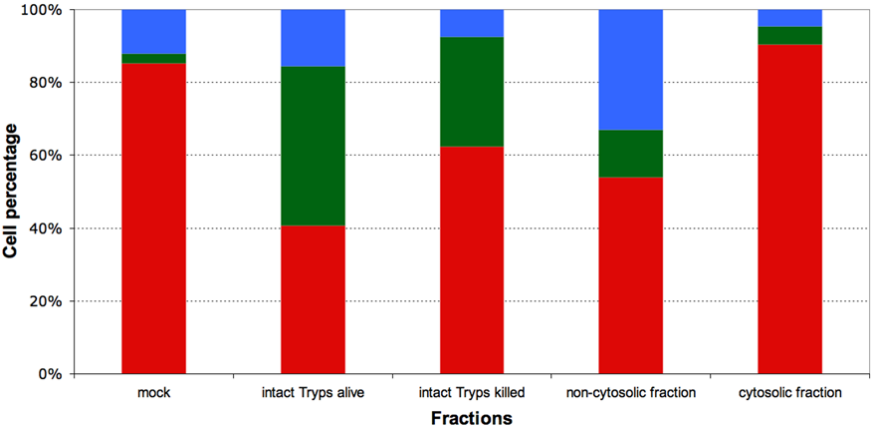
Effect of live trypanosomes, killed trypanosomes, and trypanosome fractions on lymphocyte survival. Cells were exposed for 60 min to 4 10^8^ trypanosomes per ml or to fractions corresponding to this concentration. The three cell subsets represented are normocytic and annexinV/PI double negative (red, healthy), normocytic and annexinV/PI double positive (green, early necrosis), and microcytic and annexinV/PI double positive (blue, late necrosis). Values presented are means of three independent experiments.

In study 2, mouse PBMCs were exposed to suspensions containing either live or killed trypanosomes or the cytosolic or non-cytosolic fraction of disrupted parasites (Fig. 6). The FL2/SSC dot plots obtained after 60 minutes of exposure revealed induction of the necrotic process by live parasites (43.6±1.3% [P2] and 15.6±1.5% [P3], p<0.0001), killed parasites (30.5±1.2% [P2] and 7.5±0.8% [P3], p<0.0001), and the non-cytosolic fraction (13.1±0.7% [P2] and 32.9±1.7% [P3]). In contrast, PBMC exposure to the cytosolic fraction failed to cause any significant expansion of populations P2 and P3 as compared to mock-exposed control PBMCs (5.0±0.2 vs. 2.8±0.3% [P2] and 4.7±0.4 vs. 11.7±0.3% [P3]).

**Figure 7:**
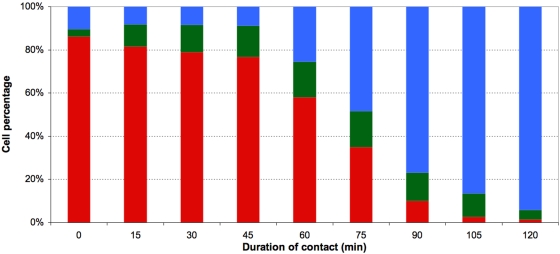
Kinetics of mouse splenocyte death induction by exposure to the non-cytosolic fraction corresponding to a parasite load of ∼4 10^8^/ml. The three cell subsets represented are normocytic and annexinV/PI double negative (red, healthy), normocytic and annexinV/PI double positive (green, early necrosis) or microcytic and annexinV/PI double positive (blue, late necrosis). Values presented are means of three independent experiments.

**Figure 8:**
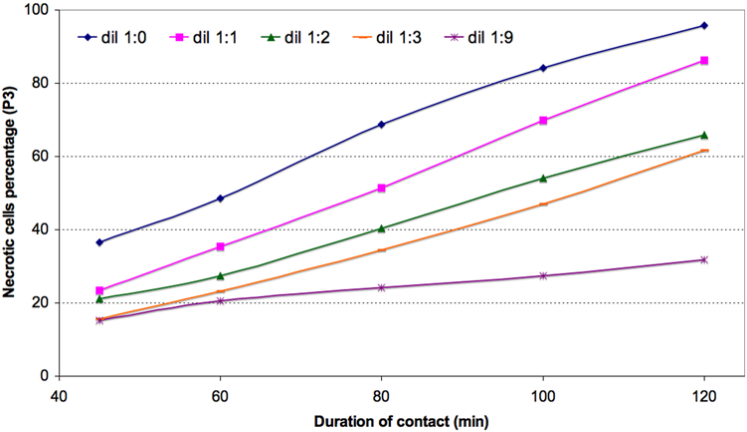
Kinetics of mouse splenocyte necrosis induction by decreasing concentrations of a stock non-cytosolic fraction titrating 8 10^8^ trypanosome equivalents per ml.

In study 3, mouse splenocytes were exposed to the non-cytosolic fraction. Induction of necrosis was again observed, and again the effect increased when the exposure time (Fig. 7) or the number of parasites added (Fig. 8) was increased. During the first 45 minutes of contact, population P2 increased in size while the size of population P3 remained stable. Later, the size of population P2 remained stable (∼15%) while population P3 increased gradually in size until finally it comprised nearly all of the exposed splenocytes (48.2±0.5 and 93.9±0.7% respectively after 75 and 120 minutes of exposure).

**Figure 9:**
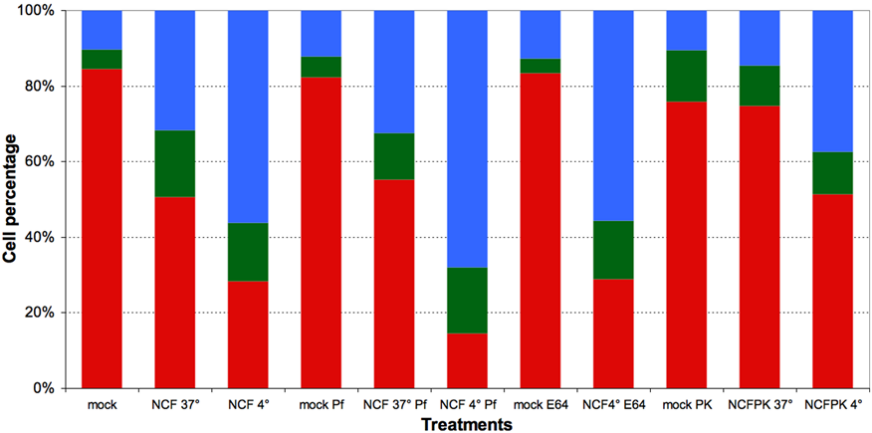
Effect on splenocyte survival of diverse pretreatments applied to the non-cytosolic trypanosome fraction (NCF). Splenocytes were exposed for 75 min to the non-cytosolic fraction corresponding to 4 10^8^ trypanosome equivalents per ml. Mock, PBS; NCF 37°C and 4°C, non-cytosolic fraction kept at 37 or 4°C respectively for 24 h prior to incorporation onto the cells; Pf, E64 and PK, treatment of NCF with Pefablock or E64 or proteinase K respectively for 24 hours prior to incorporation onto cells. The three cell subsets represented are normocytic and annexinV/PI double negative (red, healthy), normocytic and annexinV/PI double positive (green, early necrosis), and microcytic and annexinV/PI double positive (blue, late necrosis). The values presented are means of three independent experiments.

In study 4, mouse splenocytes were exposed to a non-cytosolic fraction pre-incubated at 4°C or 37°C in the presence or absence of different agents (Fig. 9). After 24 hours of incubation at 4°C or 37°C, the non-cytosolic fraction was found to have retained its ability to exert a pronecrotic effect on splenocytes, the effect being stronger after incubation at 4°C (Fig. 9, stacks 1–3). Pre-incubation in the presence of a serine protease inhibitor exacerbated the pronecrotic effect (Fig. 9, stacks 4–6), but pre-incubation in the presence of a cysteine protease inhibitor did not (Fig. 9, stacks 7–8). On the other hand, pre-incubation of the non-cytosolic fraction for 24 hours in the presence of proteinase K significantly attenuated (in the case of partial digestion at 4°C) or even abolished (in the case of total digestion at 37°) the pronecrotic effect (Fig. 9, stacks 9–11). Lastly, after a 24-hour pre-incubation in the presence of DNAse, the pronecrotic action of the fraction appeared unchanged (data not shown). Taken together, these results suggest that the pronecrotic activity is exerted by at least one protein that is not a protease and that is sensitive to serine proteases but not to cysteine proteases.

In study 5, mouse splenocytes were exposed to each of the sub-fractions obtained from the non-cytosolic fraction by successive filtrations (cut-offs at 50 and 30 kDa). The pronecrotic activity of the fractions (or the absence thereof) was determined as above. Splenocyte exposure to either retained sub-fraction failed to elicit any change as compared to mock-exposed control splenocytes (p>0.10). In contrast, exposure to the final filtrate (<30 kDa) always resulted in induction of necrosis (data not shown).

**Figure 10:**
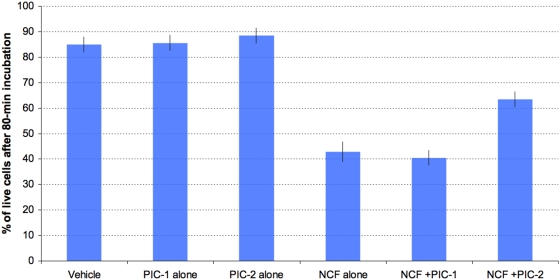
Effect of antiphosphatase incorporation on splenocyte survival upon exposition to the non-cytosolic trypanosome fraction (NCF). Splenocytes were exposed for 80 min to the non-soluble fraction corresponding to 4 10^8^ trypanosome equivalents per ml. Vehicle, PBS ; PIC-1 & -2, phosphatase inhibitor 1 and 2 respectively ; NCF, non-cytosolic trypanosome fraction. Values are means±SD of three independent experiments. Asterisks show values significantly different from T values (P<0.05).

In study 6, mouse splenocytes were exposed to the non-cytosolic fraction in the presence or absence of phosphatase inhibitor 1 or 2 (Fig. 10). Controls incubated without the non-cytosolic fraction but with either phosphatase inhibitor cocktail for 80 minutes showed no enhancement of necrosis as compared to incubation with PBS (Fig. 10, p>0.10). The pronecrotic effect expected upon exposure to the non-cytosolic fraction was confirmed. It was observed also in the presence of phosphate inhibitor cocktail 1 (Fig. 10, p<0.0001), but it was abolished in the presence of phosphate inhibitor cocktail 2 (Fig. 10, p>0.10). The complete non-cytosolic fraction and the 50-kDa filtrate both showed high phosphatase activity (data not shown), but no activity of this type was detected in the final filtrate (<30 kDa).

**Figure 11:**
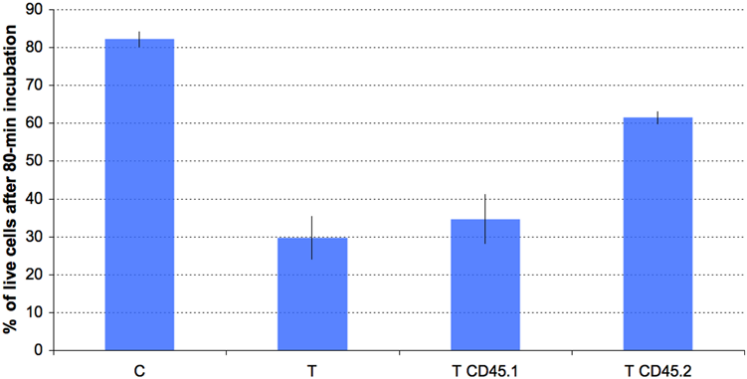
Mouse splenocyte survival upon exposure to the trypanosomal non-cytosolic fraction is increased by anti-CD45.2 but not by anti-CD45.1 mAb. Splenocytes were from C57BL/6 mice, which are homozygous for the CD45.2 genotype. C, mock-exposed splenocytes; T, T CD45.1 and T CD45.2, splenocytes exposed to the trypanosomal non-cytosolic fraction after preconditioning with either PBS, anti-CD45.1, or anti-CD45.2 mAb (15 µg/5.10^5^ cells) respectively. Values are relative fractions of annexinV/PI-double-negative (healthy) splenocytes after an 80-min exposure, as determined by flow cytometry. Values are means±SD of three independent experiments. Asterisks show values significantly different from T values (P<0.05).

**Figure 12:**
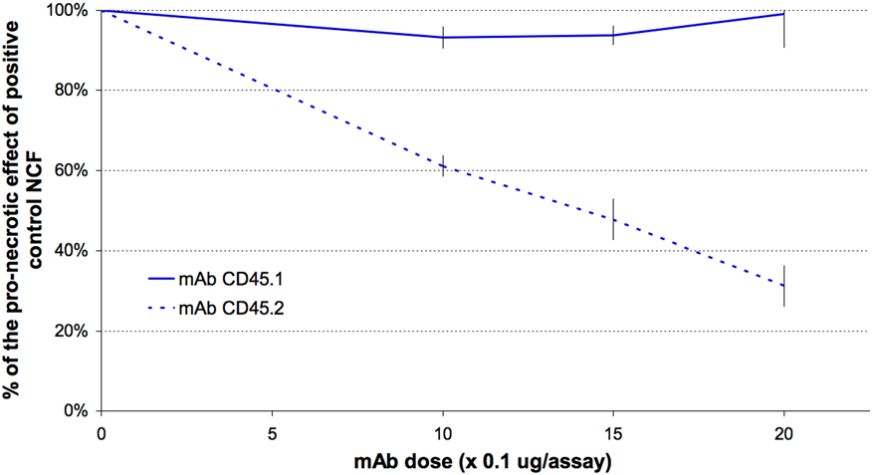
CD45 neutralization protects mouse splenocytes against the lethal effect of the trypanosomal non-cytosolic fraction in a dose-dependent way, provided the neutralizing mAb targets the epitope typical of CD45.2 rather than that characteristic of CD45.1. Splenocytes were from C57BL/6 mice, which are homozygous for the CD45.2 genotype. Values are means±SD of three independent experiments.

In study 7, incubation with anti-CD45.1 failed to abolish the pronecrotic effect of the non-cytosolic fraction. This was expected, as the epitope concerned is absent in the CD45 expressed by strain C57BL/6 (Fig. 11). In contrast, the number of PI-incorporating cells diminished significantly and in a dose-dependent manner in response to addition of anti-CD45.2 (Figs. 11–12). Histologically, addition of anti-CD45.2 did not induce leukocyte clustering.

### *In vivo* testing of non-cytosolic fraction

Whereas intravenous injection of PBS did not result in altered lymphocyte counts, administration of the non-cytosolic fraction caused a significant 49.5±10.3% drop (p<0.01, paired *t*-test) compared to preinjection values. Similarly to the observations made previously during peak parasitaemia-associated lymphopenia, mock- and non-cytosolic fraction-exposed mice showed no significant difference in their counts of circulating apoptotic and necrotic lymphocytes (p>0.05).

## Discussion

### *In vivo* studies

In this study, peripheral blood lymphocytes and their subsets were counted over the first parasitaemic wave in C57BL/6 mice inoculated with *T. evansi*. The present total and subset-specific lymphocyte counts for control mice are similar to previously reported counts [15]. The total lymphocyte count decreased sharply when the parasite load approached 10^7^/ml, reaching a nadir ∼6 hours after the peak parasitaemia and recovering quickly when parasitaemia declined. These measurements in laboratory mice confirm the observations of lymphopenia noted in most reports on trypanosomiasis cases. They suggest a possible causal link. Lymphopenia might imaginably arise through bone marrow or thymus suppression, altered lymphocyte trafficking, or through a direct pronecrotic or pro-apoptotic action of trypanosomes on lymphocytes, as recently described for the spleen marginal zone IgM+ B cell population during early onset of a *T. brucei* infection [16]. As peripheral lymphocyte counts recovered steeply after the parasite wave waned, suppression of leukopoiesis seems unlikely. Furthermore, the fact that inhibition of NO production and neutralization of TNFα exacerbated lymphocyte loss rules out the possibility that NO production or TNFα signalling might contribute to leukopenia as they contribute to anaemia [12], [17], [18]. Interestingly, the lymphopenia worsened concomitantly with increasing parasite load. This, again, suggests a link. No alterations typical of necrosis/apoptosis were detected among peripheral blood lymphocytes, even when activated caspase 3 was sought by western blotting in buffy coats from highly parasitaemic mice (>10^8^ trypanosomes/ml, data not shown). Yet as altered lymphocytes most likely undergo rapid sequestration *in vivo*, the possible triggering of apoptosis or necrosis by trypanosomes was sought *in vitro*. As the lymphocyte subset ratios were significantly altered too, it might be that the process engaged or its kinetics differs between subsets. Moreover, while T lymphocyte counts showed a continuous decrease, B lymphocyte counts showed a biphasic change, with an early short-lived increase (52 h pi) followed by a dramatic abatment, which suggests that the expected B cell expansion duly occurred but was not sustainable in face of the rising parasitaemia.

### *In vitro* studies

After purification and a 60-min incubation at 37°C in RPMI, about 20% of the mock-exposed PBMCs displayed spontaneously occurring regressive alterations typical of either necrosis (∼15%) or apoptosis (∼5%). An identical profile was observed when the cells were incubated in the presence of trypanosome-free plasma from parasitaemic, lymphopenic mice. These first *in vitro* results suggest (i) that the purification process itself affected about one-fifth of the cells and (ii) that trypanosomes do not secrete a soluble factor capable of causing necrosis or apoptosis of circulating lymphocytes and thus explaining the lymphopenia. Addition of live trypanosomes to the PBMC-containing medium did not cause apoptosis but it did lead to a concentration- and contact-time-dependent increase in the number of cells undergoing necrosis. To our knowledge, there exists no previous data establishing the existence of trypano-dependent necrosis, although it is known that *T. cruzi* [19], [20], [21] and *T. brucei* [16], [22] can cause apoptosis. The present study thus suggests that induction of necrosis by the parasite itself is at least partially responsible for the lymphopenia occurring concomitantly with parasitaemia peaks. When PBMCs were incubated with killed trypanosomes, the effect was similar, albeit less drastic. This suggests that parasite motion may favour the pronecrotic activity, probably by maximizing parasite-lymphocyte contacts. On the other hand, only the non-cytosolic fraction of the parasites was able to reproduce the pronecrotic effect exerted by whole trypanosomes. Taken together, these results suggest the existence of a parasite-membrane-associated pronecrotic factor whose activity depends on direct physical contact with lymphocytes. Furthermore, it is known that the phospholipases released during the parasite fractioning procedure cleave part of the «variable surface glycoproteins» (VSGs), which are thus abundant in both fractions studied [23], [24]. It therefore seems likely that VSGs do not intervene in the necrotic process. By subjecting the non-cytosolic fraction to various pre-treatments we have refined our picture of the pronecrotic factor: firstly, it is resistant to protease inhibitors and to cysteine proteases but sensitive to serine proteases. This suggests that it consists of one or several non-cytosolic, and hence probably membrane-associated, proteins that are not proteases and that possess the cleavage site used by serine proteases. Secondly, our data concerning the sub-fractions obtained from the non-cytosolic fraction by successive filtrations show that the molecular mass of the pronecrotic factor is below 30 kDa; this again suggests that VSGs are not concerned [25]. Finally, intravenous injection of the <30 kDa non-cytosolic subfraction duly reproduced a dramatic lymphopenic trough, which demonstrates that the pronecrotic factor works *in vivo* as well.

Our results obtained with one phosphatase inhibitor added to the medium reveal that *T. evansi*-associated necrosis is PTPase dependent. As the inhibitor used (sodium orthovanadate) is cell permeable, the PTPase concerned could be parasitic or lymphocytic. The fact that the final filtrate obtained by parasite fractionation (<30 kDa) still exerts pronecrotic activity without showing any phosphatase activity rules out the possibility that the PTPase concerned derives from the parasite. This leads quite naturally to the hypothesis that the lymphocyte-membrane-associated PTPase CD45 might be the PTPase in question, given its status as a prototype PTPase and its abundance at the lymphocyte surface [26]. This hypothesis is corroborated by our observation that addition of an anti-murine-CD45 mAb caused a 50% decrease in the number of cells undergoing necrosis as a result of exposure to the various non-cytosolic trypanosome fractions. To our knowledge, this is the first demonstration of such an interaction between lymphocyte-associated CD45 and a trypanosomal-membrane-associated protein ligand. The known regulatory functions of CD45 concern the activation threshold of the TCR-dependent signal, the magnitude and nature of cytokinic responses, and lymphocyte fate [27]. For instance, CD45-null mice show increased cell death [28], and ligation of CD45 by antibodies or galectin leads to cell death [29], [30], [31], [32]. The present demonstration of CD45 involvement in *T. evansi*-dependent lymphotoxicity thus points to a possible manipulation of the host's cytokinic and adaptive responses by trypanosomes via CD45. The large extracellular domain of CD45 is notable for its highly glycosylated and sialylated state, which varies according to the inclusion or exclusion of alternatively spliced exons 4, 5, and 6. The resulting isoforms are specific not only to the haematopoietic cell type, but also to the stage of differentiation and activation of the cell. This observation might explain why only a fraction of the circulating PBMCs showed PI incorporation upon exposure to trypanosome extracts. It has also been suggested that these alternative isoforms might interact with unique ligands, but to date, no isoform-specific ligand has been convincingly identified. In the present case, the leukotoxic trypanosomal ligand concerned remains to be determined. All we can say about it is that it is a serine-protease-sensitive membrane protein with a molecular mass of less than 30 kDa.
